# Platelet-rich plasma attenuates the severity of joint capsule fibrosis following post-traumatic joint contracture in rats

**DOI:** 10.3389/fbioe.2022.1078527

**Published:** 2023-01-04

**Authors:** Yuxin Zhang, Zengguang Wang, Chenyu Zong, Xiaoding Gu, Shuai Fan, Lili Xu, Bin Cai, Shenji Lu

**Affiliations:** ^1^ Department of Rehabilitation Medicine, Shanghai Ninth People’s Hospital, Shanghai Jiao Tong University School of Medicine, Shanghai, China; ^2^ Department of Oral Surgery, Shanghai Ninth People’s Hospital, Shanghai Jiao Tong University School of Medicine, College of Stomatology, Shanghai Jiao Tong University, National Center for Stomatology, National Clinical Research Center for Oral Diseases, Shanghai Key Laboratory of Stomatology, Shanghai, China; ^3^ Department of Rehabilitation Medicine, Huangpu Branch, Shanghai Ninth People’s Hospital, Shanghai Jiao Tong University School of Medicine, Shanghai, China; ^4^ Shanghai Key Laboratory of Orthopedic Implants, Department of Orthopaedic Surgery, Shanghai Ninth People’s Hospital, Shanghai Jiao Tong University School of Medicine, Shanghai, China; ^5^ Department of Orthopedics, Affiliated Hospital of Nantong University, Nantong, China

**Keywords:** platelet-rich plasma, post-traumatic joint contracture, joint capsule fibrosis, transforming growth factor-β1, fibroblasts

## Abstract

**Background:** Post-traumatic joint contracture (PTJC) mainly manifests as excessive inflammation leading to joint capsule fibrosis. Transforming growth factor (TGF)-β1, a key regulator of inflammation and fibrosis, can promote fibroblast activation, proliferation, migration, and differentiation into myofibroblasts. Platelet-rich plasma (PRP) is considered to have strong potential for improving tissue healing and regeneration, the ability to treat joint capsule fibrosis remains largely unknown.

**Methods:** In this study, we aimed to determine the antifibrotic potential of PRP *in vivo* or *in vitro* and its possible molecular mechanisms. The TGF-β1-induced primary joint capsule fibroblast model and rat PTJC model were used to observe several fibrotic markers (TGF-β1, α-SMA, COL-Ⅰ, MMP-9) and signaling transduction pathway (Smad2/3) using histological staining, qRT-PCR and western blot.

**Results:** Fibroblasts transformed to myofibroblasts after TGF-β1 stimulation with an increase of TGF-β1, α-SMA, COL-Ⅰ, MMP-9 and the activation of Smad2/3 *in vitro*. However, TGF-β1-induced upregulation or activation of these fibrotic markers or signaling could be effectively suppressed by the introduction of PRP. Fibrotic markers’ similar changes were observed in the rat PTJC model and PRP effectively reduced inflammatory cell infiltration and collagen fiber deposition in the posterior joint capsule. Interestingly, HE staining showed that articular cartilage was degraded after rat PTJC, and PRP injection also have the potential to protect articular cartilage.

**Conclusion:** PRP can attenuate pathological changes of joint capsule fibrosis during PTJC, which may be implemented by inhibiting TGF-β1/Smad2/3 signaling and downstream fibrotic marker expression in joint capsule fibroblasts.

## Introduction

Post-traumatic joint contracture (PTJC) of the knee is a common complication of joint injury and immobilization. Loss of range of motion (ROM) of the affected joint reduces normal mobility and quality of life (Hildebrand et al., 2004). Non-surgical and surgical treatments have been used to prevent contractures. However, the mechanism and precautions of PTJC are still being explored (Campbell et al., 2014; Chen et al., 2019a). Joint contracture results from intra-articular adhesion formation and joint capsular fibrosis due to inflammation. During the process, posterior joint capsule inflammation, thickness, extracellular matrix (ECM) deposition, collagen hyperplasia are represented (Lake et al., 2016). As the main cell types of joint capsules, fibroblasts play a key role in ECM synthesis and remodeling, as well as inflammation and immune regulation (Van Linthout et al., 2014). Under pathological conditions such as injury or inflammation, transforming growth factor (TGF)-β1, a key regulator of inflammation and fibrosis, is rapidly secreted by immune cells to promote fibroblast activation, proliferation, migration, and differentiation into myofibroblasts (Sasabe et al., 2017; Zhou et al., 2020), which is a key step in the physiological reparative response to tissue damage (Vallee and Lecarpentier, 2019; Weiskirchen et al., 2019). Activated myofibroblasts highly express α-smooth muscle actin (α-SMA) and secrete higher amounts of collagen and other fibrotic factors. However, TGF-β1 levels are chronically elevated in patients and animals with PTJC, and blocking TGF-β1 expression and downstream signal pathway activation during joint trauma are effective treatment strategy to reduce the severity of joint capsule fibrosis (Hildebrand et al., 2007; Mattyasovszky et al., 2016).

Platelet-rich plasma (PRP) is increasingly used in the treatment of musculoskeletal diseases, however, the effectiveness and mechanism have not yet been clearly demonstrated. It is prepared after blood collection and contains a high platelet concentration, thus, the product contains many pro-inflammatory and anti-inflammatory cytokines, which may lead to various effects. PRP has the potential to improve tissue healing and regeneration (Sanchez et al., 2014; Etulain, 2018). A large number of clinical studies demonstrated that PRP could relieve pain or osteoarthritis, plantar fasciitis, and tendinosis upon its anti-inflammatory effect (Kuffler, 2019). However, the antifibrotic potential of PRP remains controversial. PRP has been reported to have a preventive effect on stricture formation in a rat urethral injury model by its effect on collagen synthesis (Tavukcu et al., 2018). In contrast, Lee et al. demonstrated that PRP could promote the process of meniscal fibrosis *in vivo* (Lee et al., 2016). However, few studies have focused on the ability of PRP to treat joint capsule fibrosis (Dunham et al., 2019).

In this study, we aimed to determine the antifibrotic potential of PRP *in vitro* and *in vivo* and its possible molecular mechanisms using the TGF-β1-induced primary joint capsule fibroblast model and rat PTJC model by evaluating several fibrotic markers and signaling transduction pathways.

## Materials and methods

### Animals

Adult male Sprague Dawley (SD) rats (180–220 g) were purchased from Shanghai SIPPR-Bk Lab Animal Co., Ltd. and maintained in specific pathogen-free laboratory animal facilities of Shanghai Ninth People’s Hospital, and housed in individual cages at room temperature (23 ± 1°C), relative humidity, and a 12 h light/dark cycle with free access to food and water. All animal experiments complied with the National Institutes of Health Guide for the Care and Use of Laboratory Animals and were reviewed and approved by the Institution of Animal Care and Use Committee (IACUC) of the Ninth People’s Hospital Affiliated to Shanghai Jiao Tong University School of Medicine.

### Platelet-rich plasma preparation

First, 9 ml blood from the artery of each donor animal (10-week male rat) was pumped and then mixed with 1 ml of 3.8% sodium citrate. An extra 1 ml blood sample was set apart to determine the concentration of platelets. Subsequently, the blood samples were centrifuged at 200 g for 10 min at 4°C. The supernatant and the interlayer of the buffy coat were transferred to another centrifuge tube and centrifuged at 1,000 g for 10 min at 4°C. A total of 3/4 of the supernatant was discarded, PRP was achieved through resuspension of the remaining sample, and the concentration of platelets was approximately 2 × 10^6^/µl. PRP was stored at -80°C and was activated by the addition of 10% CaCl_2_ before use.

### Cell culture and treatment

Posterior joint capsules were obtained from the knee joints of healthy SD rats (male, 4 weeks old) after euthanization and subsequently washed with Dulbecco’s minimum essential medium (DMEM). The tissue fragments were cut into 2–3 mm, arranged in a 6-well culture plate containing 1 ml DMEM supplemented with 1% penicillin/streptomycin and 10% fetal bovine serum (FBS), and cultured in an incubator at 5% CO_2_ and 37°C. The medium was changed every couple of days. Fibroblasts started to migrate from the subsections after 3–5 days and tissues were removed on approximately the seventh day when the culture reached 90% confluence. Primary joint capsule fibroblasts were cultured to passages 2 to 4 and identified by cells exhibiting a characteristic morphology and positive staining for the fibroblast marker vimentin (Abcam) before used in subsequent experiments.

### TGF-β1 inducing

Fibroblasts were cultured in serum-low medium (basic medium with 0.3% FBS) for 16 h and then induced with 10 ng/ml TGF-β1 for 24 h. The groups of experiments were set as follows: 1) CON: fibroblasts with DMEM; 2) PRP: fibroblasts with DMEM and PRP (1:50); 3) TGF-β1: fibroblasts with DMEM and 10 ng/ml TGF-β1; and 4) TGF-β1 + PRP: fibroblasts with DMEM, 10 ng/ml TGF-β1, and PRP (1:50) (Sassoli et al., 2018).

### Cell viability assay

Fibroblasts were plated in a 96-well plate at a density of 1× 10^5^ cells/mL overnight and subsequently treated with TGF-β1 (0, 0.5, 1, 5, 10 ng/ml) for 24 h. Then, 10 µl of Cell Counting Kit-8 (CCK-8; Dojindo) was added to the cells and incubated for an additional 2 h at 37°C. Cell viability was measured using a spectrophotometer by detecting absorbance of each well at 450 nm. Assays were performed three times using triplicate wells.

### Rat post-traumatic joint contracture model

As previously described (Zhang et al., 2021a; Zhang et al., 2021b), rats were anaesthetized *via* intraperitoneal injection of sodium pentobarbital (50 mg/kg) and placed in a supine position for surgery. The knee joint of the right hind leg underwent a midline skin incision and a lateral parapatellar arthrotomy was performed. The patella was moved medially and the femoral condyle was exposed by flexing the knee joint. Two 1.5 × 1.5-mm cortical windows were made from non-articulating cartilaginous regions of the lateral and medial femoral condyles using a 1.5-mm drill bit. The anterior and posterior cruciate ligaments were incised, and the posterior joint capsule was disrupted by hyperextension of the knee at −45°. The right knee was immobilized at 135°- flexion with a 0.5-mm steel wire passing through the holes made by a drill bit in the proximal femur and the distal tibia and buckled at the subcutaneous medial knee joint. Finally, after patellofemoral joint reduction, the muscles and skin were sutured with silk threads. For drug delivery, activated PRP was injected into the joint cavity after sutured. After surgery, rats were allowed unrestricted daily activity in cages. Rats were euthanized at 0 days and 4 weeks, internal fixation was removed, and the knee extension ROM was measured within 15 min of euthanasia. Affected knee joint and posterior joint capsules were collected for subsequent analysis.

### Histological assessment

After euthanization using an overdose of sodium pentobarbital, rat knee joints and posterior capsule tissues were removed, fixed with 4% paraformaldehyde, decalcified in 10% EDTA, and then embedded in paraffin. Samples were embedded in paraffin and cut into 4-μm thick sections. Hematoxylin-eosin (HE) staining and Masson trichrome staining were used for qualitative observation. The tissues were deparaffinized and hydrated in distilled water and then covered with hematoxylin and eosin, respectively, by using an HE staining kit (Abcam). Masson staining was conducted using tissue covered by Weigert iron hematoxylin, Biebrich scarlet-acid fuchsin and aniline blue (Sigma-Aldrich) according to the manufacturer’s instructions.

### Immunohistochemistry

Posterior joint capsule tissues were removed and quickly frozen for subsequent immunohistochemical staining of TGF-β1, α-SMA, COL-Ⅰ, and matrix metalloproteinase (MMP)-9 (Servicebio). Briefly, the slides were incubated with primary antibodies overnight at 4°C. Secondary goat anti-rabbit antibodies were applied to the slides after washing at room temperature for 1 h. DAB solution was applied after the washing step and then incubated in the dark for 30 min followed by counterstaining with hematoxylin. All images were captured using a Zeiss LSM710 confocal microscope (Zeiss). 

### Quantitative reverse transcription polymerase chain reaction

Total RNA of was extracted with TRIzol Reagent (Invitrogen) according to the manufacturer’s instructions and was then reverse transcribed using the Omniscript Reverse Transcription Kit (QIAGEN). qRT-PCR was carried out on a real-time PCR system (Applied Biosystems) using SYBR® Premix Ex Taq™ (Takara Bio). *Gapdh* was used as a reference gene and calculated the relative levels using 2^−ΔΔCt^. The primers were designed and synthesized by Sangon Biotech (Shanghai, China) as follows: *Gapdh*: forward primer 5ʹ-ACA GCA ACA GGG TGG TGG AC-3ʹ, reverse primer 5ʹ-TTT GAG GGT GCA GCG AAC TT-3ʹ; *a-sma*: forward primer 5ʹ-ATC GTC CAC CGC AAA TGC-3ʹ, reverse primer 5ʹ-AAG GAA CTG GAG GCG CTG-3ʹ; *Tgf-β1*: forward primer 5ʹ-AGC AAC AAT TCC TGG CGT TAC-3ʹ, reverse primer 5ʹ-TGT ATT CCG TCT CCT TGG TTC A-3ʹ; *Col-Ⅰ*: forward primer 5ʹ-TGT ATC ACC AGA CGC AGA AGT-3ʹ, reverse primer 5ʹ-ACC AGG AGG ACC AGG AAG T-3ʹ; *Mmp-9*: forward primer 5ʹ-CCT ACT GCT GGT CCT TCT GAG-3ʹ, reverse primer 5ʹ-TGG CTT CCT CCG TGA TTC G-3ʹ.

### Western blot

Proteins were extracted from the cells and the posterior capsules. Samples were lysed in RIPA buffer containing protease and phosphatase inhibitors. After centrifugation, protein concentrations in the supernatants were determined using the BCA Protein Assay Kit (Beyotime). Western blots was performed as previously described (Zhang et al., 2019a; Zhang et al., 2021a). Briefly, proteins were separated by electrophoresis on NuPAGE® 4–12% Bis-Tris Gel (Invitrogen) for 40 min at 200 V and transferred onto polyvinylidene difluoride (PVDF) membranes (Invitrogen). The membranes were blocked with 5% skim milk for 30 min on a rotary shaker at room temperature. They were then incubated overnight at 4°C with the following primary antibodies: β-actin (ProteinTech, 1:5000), TGF-β1 (Abcam, 1:1000), α-SMA (Abcam, 1:1000), COL-Ⅰ (Abcam, 1:1000), MMP-9 (Abcam, 1:1000), Smad2/3 (Abcam, 1:1000), and p-Smad 2/3 (Abcam, 1:1000). Secondary antibodies included Goat anti-Rabbit IgG (H + L) (DyLight 800 4X PEG) (Invitrogen, 1:20000) and Goat anti-Mouse IgG (H + L) (DyLight 680) (Invitrogen, 1:20000). The fluorescent signals were determined with an Odyssey imaging system (Li-Cor, Lincoln, NE, United States). Relative protein expression levels were expressed as the ratio of the band intensity of the target protein to that of β-actin.

### Statistical analysis

All results are expressed as the mean ± standard deviation after analysis using SPSS 22.0 statistical software (SPSS Inc., Chicago, IL, United States). Parametric data were analyzed *via* Student’s t-test or one-way analysis of variance (ANOVA) followed by Tukey’s *post hoc* analysis for comparisons between two groups. Calculations were performed using GraphPad Prism software 4.0 (GraphPad, CA, United States). A level of *p* < 0.05 was considered statistically significant.

## Results

### Platelet-rich plasma suppressed TGF-β1-induced fibrotic markers expression of joint capsule fibroblasts at the gene level *in vitro*

**FIGURE 1:**
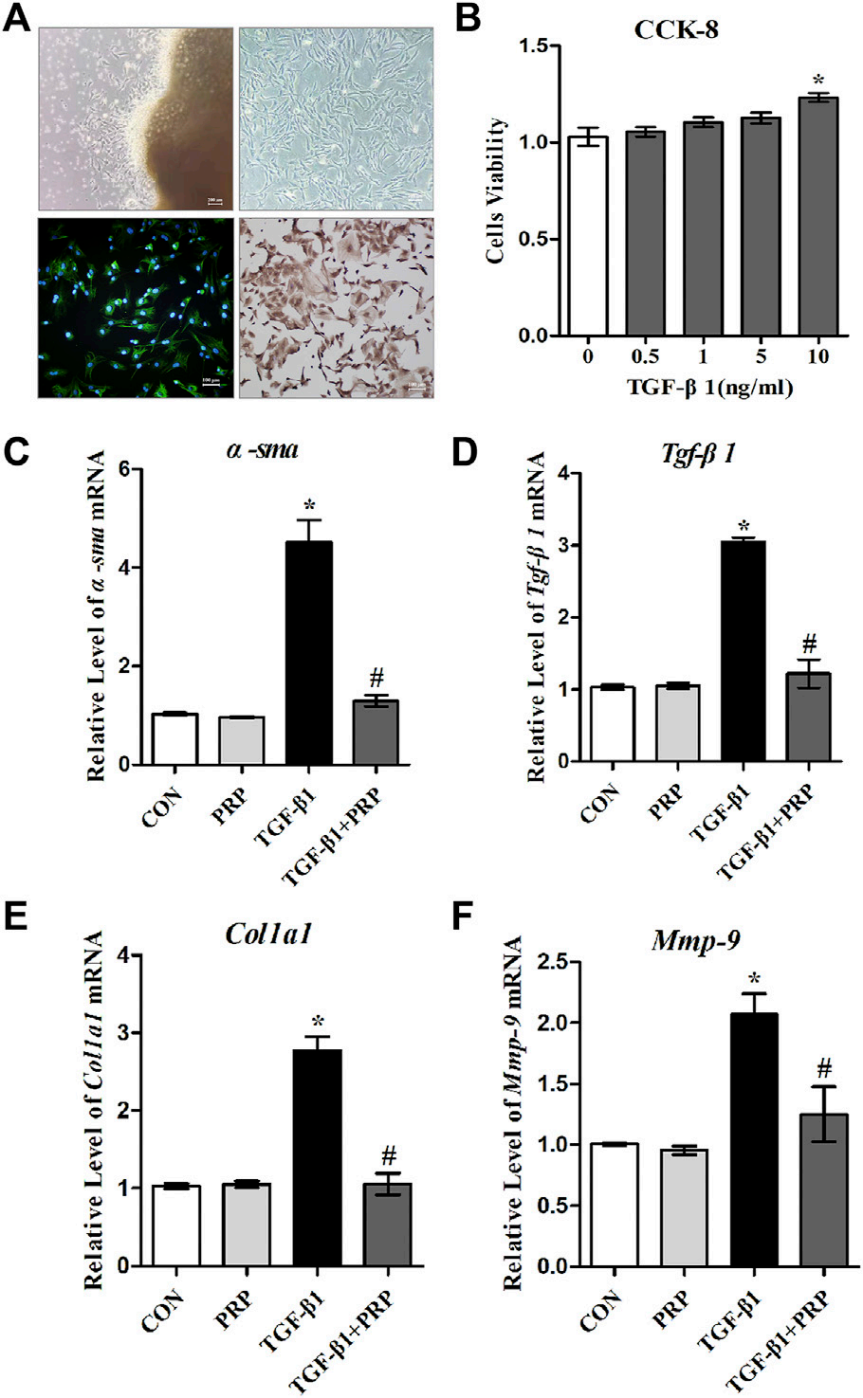
Effects of PRP on the mRNA expression of TGF-β1-induced fibrotic marker. **(A)** Primary fibroblasts were cultured and verified by immunofluorescence and immunohistochemical staining with vimentin as a marker. **(B)** Cell viability measured by CCK-8 following different concentrations of TGF-β1 treatment (0, 0.5, 1, 5, 10 ng/ml). **(C–F)** The mRNA expression of α-SMA **(C)**, TGF-β1 **(D)**, COL-Ⅰ **(E)**, MMP-9 **(F)** in fibroblasts in response to 10 ng/ml TGF-β1 combined with PRP treatment was determined by qRT-PCR. Error bars represent standard deviation. *p < 0.05 compared with the CON group. #*p* < 0.05 compared with TGF-β1 group.

Fibroblast abnormal activation is a critical process in joint capsule fibrosis. To determine the effect of PRP on fibroblast abnormal activation, we obtained primary joint capsule fibroblasts from the posterior joint capsule of 4-week-old rat knee joints (Figure 1A). Numerous studies have reported that TGF-β1 promotes the differentiation of fibroblasts into myofibroblasts, collagen hyperplasia, and ECM protein accumulation (Sasabe et al., 2017; Vallee and Lecarpentier, 2019; Weiskirchen et al., 2019; Zhou et al., 2020). As shown by CCK-8, TGF-β1 slightly enhanced fibroblast cell viability (Figure 1B), indicating that this concentration range was applicable to subsequent experiments. Therefore, we selected 10 ng/ml TGF-β1 to establish an *in vitro* fibroblast abnormal activation model and explored whether PRP inhibits activated fibroblast phenotypic transformation. The mRNA expression levels of fibrotic markers (*α-sma*, *Tgf-β1*, *Col-Ⅰ*, and *Mmp-9*) were determined by qRT-PCR (Figures 1C–F). The results showed that inactive joint capsule fibroblasts expressed very low levels of fibrosis related genes. After incubation for 24 h with 10 ng/ml TGF- β 1, the mRNA expression of fibrotic markers significantly increased, which was strongly inhibit by PRP. Interestingly, PRP alone did not significantly change the expression of fibrotic markers compared to the CON group. These results indicated that PRP suppressesed TGF-β1-induced fibrotic marker expression in joint capsule fibroblasts at the gene level *in vitro*.

### Platelet-rich plasma suppressed TGF-β1-induced fibrotic marker expression in joint capsule fibroblasts at the protein levels *in vitro*

**FIGURE 2:**
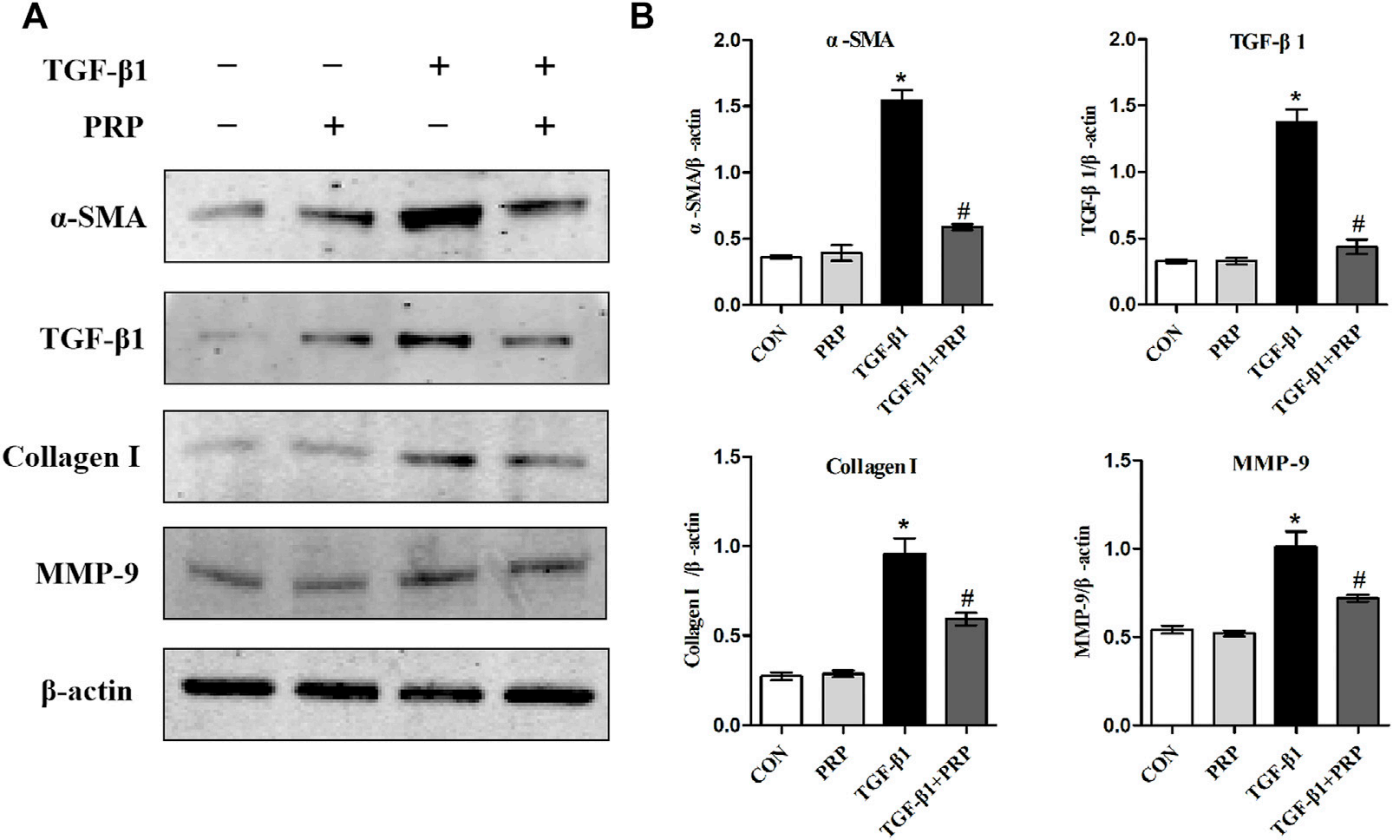
Effects of PRP on the protein expression of TGF-β1-induced fibrotic marker. **(A)** Western blot analysis of fibrosis-associated proteins α-SMA, TGF-β1, COL-Ⅰ, MMP-9 after fibroblasts treated with 10 ng/ml TGF-β1 combined with PRP. **(B)** The bar graphs represent the relative expression of these proteins after normalization to β-actin. Error bars represent standard deviation. *p < 0.05 compared with the CON group. #*p* < 0.05 compared with TGF-β1 group.

Based on the observation that PRP suppresses the transformation of joint capsule fibroblasts to myofibroblasts induced by TGF-β1 and reduces the mRNA expression of *α-sma*, *Tgf-β1*, *Col-Ⅰ*, and *Mmp-9*, we hypothesized that PRP may also suppress fibrotic markers at the protein level. Consistent with the qRT-PCR results, western blot showed that the expression of α-SMA, TGF-β1, COL-Ⅰ, and MMP-9 was significantly increased after 10 ng/ml TGF-β1 treatment. Co-incubation with PRP reversed the TGF-β1-induced fibrosis process (Figure 2). These results indicated that PRP suppressed TGF-β1-induced fibrotic marker expression in joint capsule fibroblasts at the protein level *in vitro*.

### Platelet-rich plasma inhibited the phosphorylation of Smad2/3 induced by TGF-β1 *in vitro*

**FIGURE 3:**
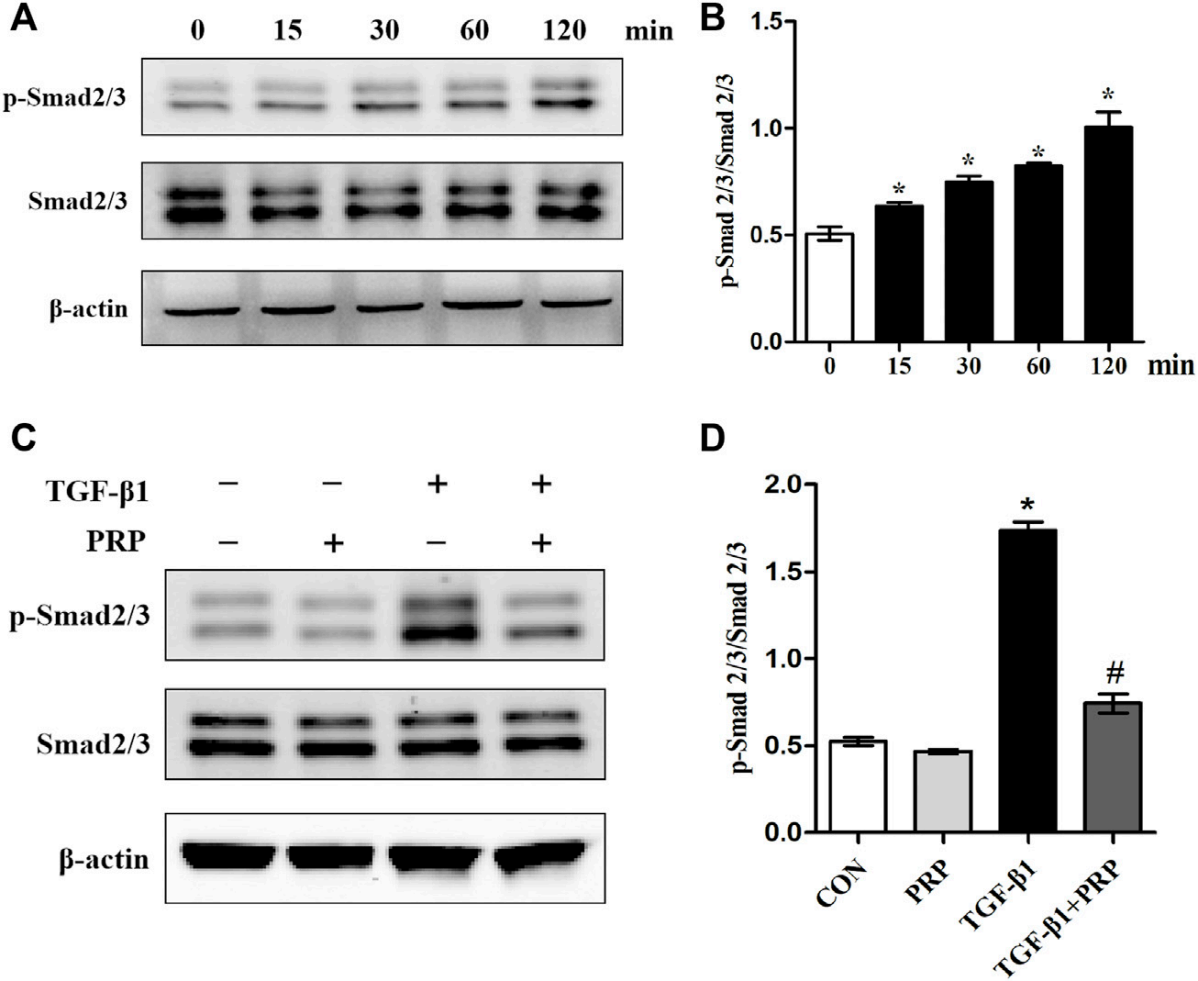
Effects of PRP on the activation of Smad2/3 signaling. **(A)** Western blot analysis the activation of fibrosis-associated signaling Smad2/3 after fibroblasts treated with 10 ng/ml TGF-β1 for 0, 15, 30, 60, and 120 min, respectively. **(B)** The bar graphs represent the relative expression of pSmad2/3/Smad2/3. *p < 0.05 compared with the 0 group. **(C)** Western blot analysis the activation of fibrosis-associated signaling Smad2/3 after fibroblasts treated with 10 ng/ml TGF-β1 combined with PRP. **(D)** The bar graphs represent the relative expression of pSmad2/3/Smad2/3. Error bars represent standard deviation. *p < 0.05 compared with the CON group. #*p* < 0.05 compared with TGF-β1 group.

**FIGURE 4:**
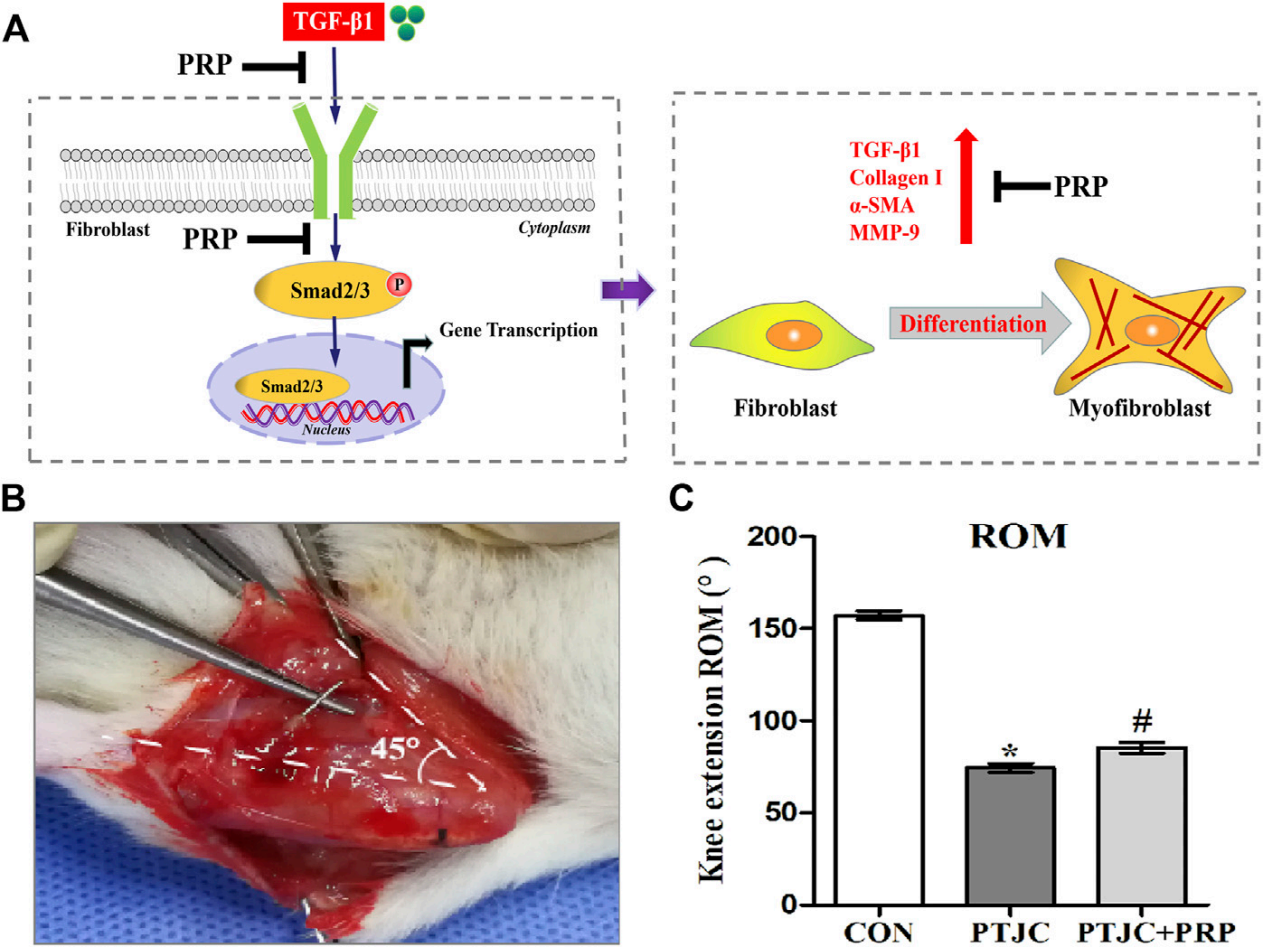
PRP inhibited the process of fibrosis. **(A)** Pattern diagram of PRP inhibited TGF-β1-induced fibrosis. TGF-β1 caused phosphorylation of Smad2/3, up-regulating expression of TGF-β1, COL-Ⅰ, α-SMA, MMP-9 and promoted fibroblasts transforming into myofibroblasts. PRP inhibited TGF-β1-induced fibrosis. **(B)** Schematic of rat knee joint post-traumatic immobilization. **(C)** Measurement of extension ROM of the affected knee joint. Error bars represent standard deviation. *p < 0.05 compared with the CON group. #*p* < 0.05 compared with PTJC group.

Considering that Smad2/3 is the major signal transduction pathway responsible for mediating profibrotic effect of TGF-β1, we therefore evaluated whether PRP exerts its antifibrotic effect through the suppression of the Smad2/3 signaling pathway. As shown in Figure 3A, western blot showed that Smad2/3 in joint capsule fibroblasts was quickly induced 15 min after stimulation with 10 ng/ml TGF-β1 and remained continuously activated for 120 min. The addition of PRP to TGF-β1-conditioned medium led to a significant decrease in Smad2/3 phosphorylation levels (Figure 3C). These results indicate that PRP suppressed TGF-β1-induced transformation of joint capsule fibroblasts to myofibroblasts and fibrotic marker expression possibly by blocking the TGF-β1/Smad2/3 pathway (Figure 4A).

### Platelet-rich plasma reduced fibrotic marker expression in posterior joint capsule following post-traumatic joint contracture *in vivo*

**FIGURE 5:**
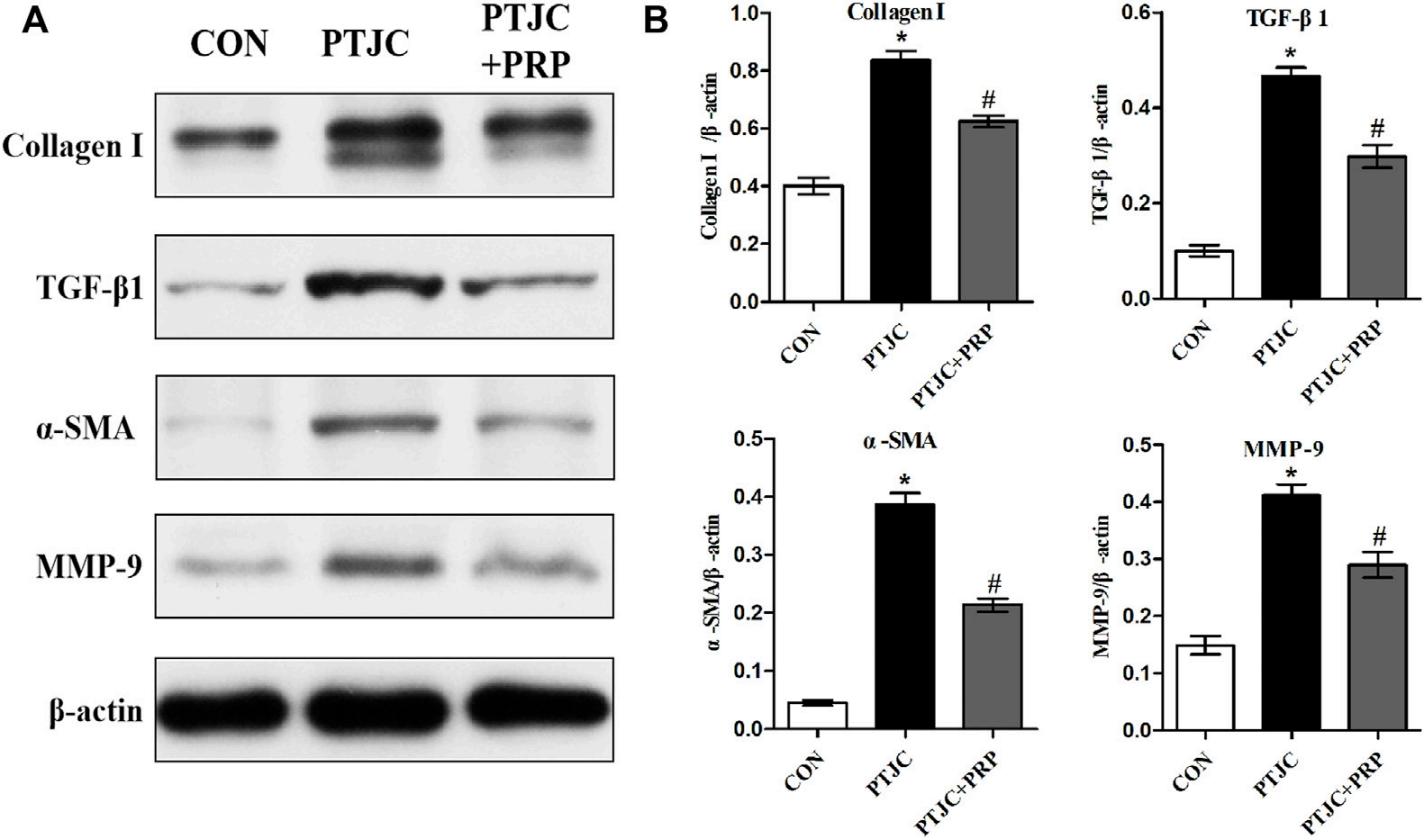
Expression of fibrosis-associated proteins in the posterior joint capsule were assessed *via* western blot. **(A)** The western blot analysis of COL-Ⅰ, TGF-β1, α-SMA, MMP-9 in the posterior joint capsule. **(B)** The bar graphs represent the relative expression of these proteins after normalization to β-actin. Error bars represent standard deviation. *p < 0.05 compared with the CON group. #*p* < 0.05 compared with PTJC group.

**FIGURE 6:**
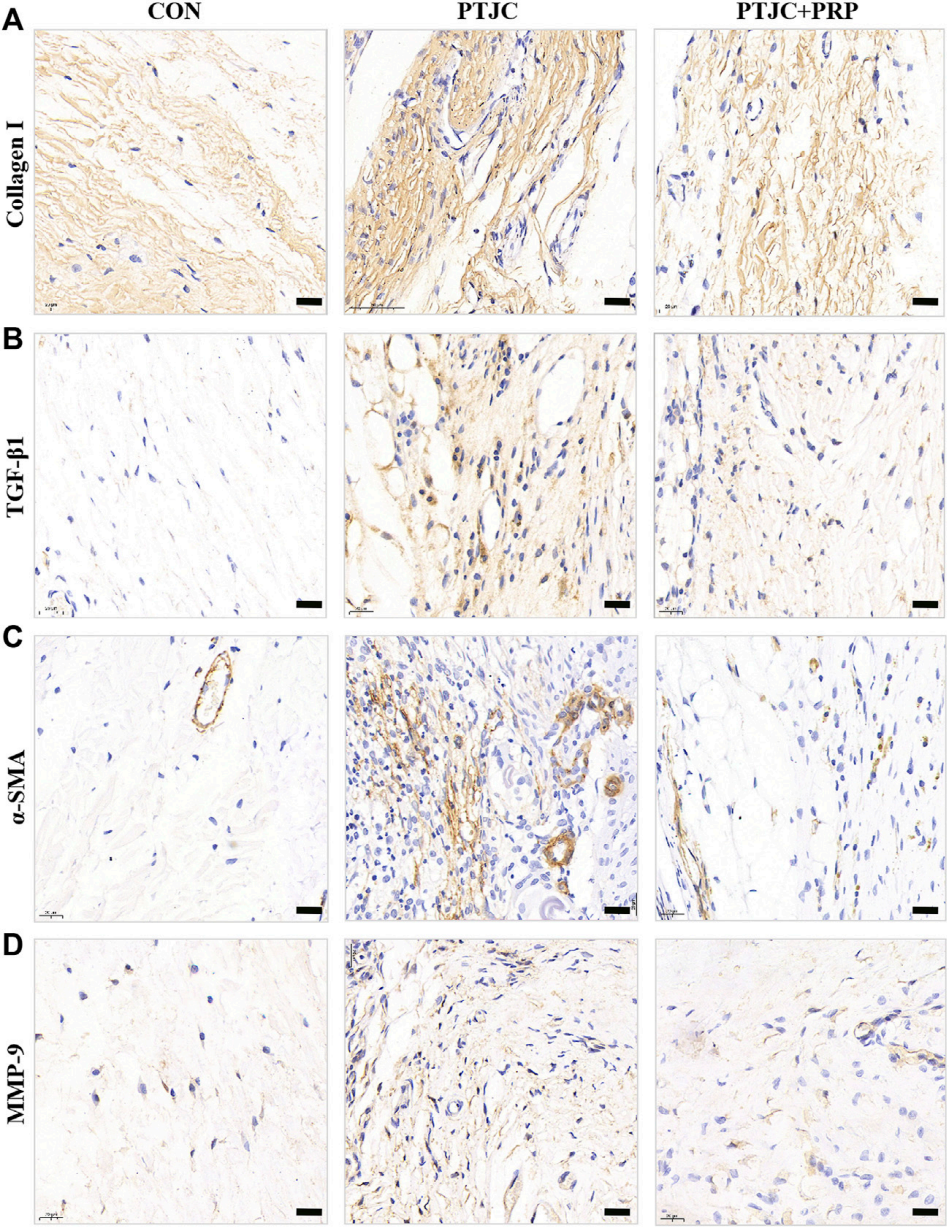
Expression of fibrosis-associated proteins in the posterior joint capsule were assessed *via* immunohistochemical. **(A–D)** The immunohistochemical staining of COL-Ⅰ **(A)**, TGF-β1 **(B)**, α-SMA **(C)**, MMP-9 **(D)** in the posterior joint capsule. Scale bars, 50 μm.

To examine whether PRP can inhibit the process of joint capsule fibrosis after PTJC *in vivo*, we established the PTJC model as previously described (Figure 4B) and measured knee extension ROM after myotomies of the trans-articular muscles. The results showed that the normal knee extension ROM was 159.9 ± 1.3° and was reduced to 73.8 ± 3.2° at 4 weeks postinduction of PTJC, while the group of rats that received an injection of PRP was increased to 85.5 ± 5.1° (Figure 4C). In addition, we further checked the expression level of fibrosis-related proteins in the rat posterior joint capsule by western blot (Figure 5) and immunohistochemistry staining (Figure 6). Consistent with the observation results of *in vitro* cell experiments, the expression of α-SMA, TGF-β1, COL-Ⅰ, and MMP-9 was significantly induced in the posterior joint capsule following PTJC, which was effectively reversed by PRP injection at the lesion sites, but was still higher than that of the CON group. These data indicated that PRP can reduce fibrotic marker expression both *in vitro* and *in vivo*.

### Platelet-rich plasma attenuated joint capsule inflammation and fibrosis following post-traumatic joint contracture *in vivo*

**FIGURE 7:**
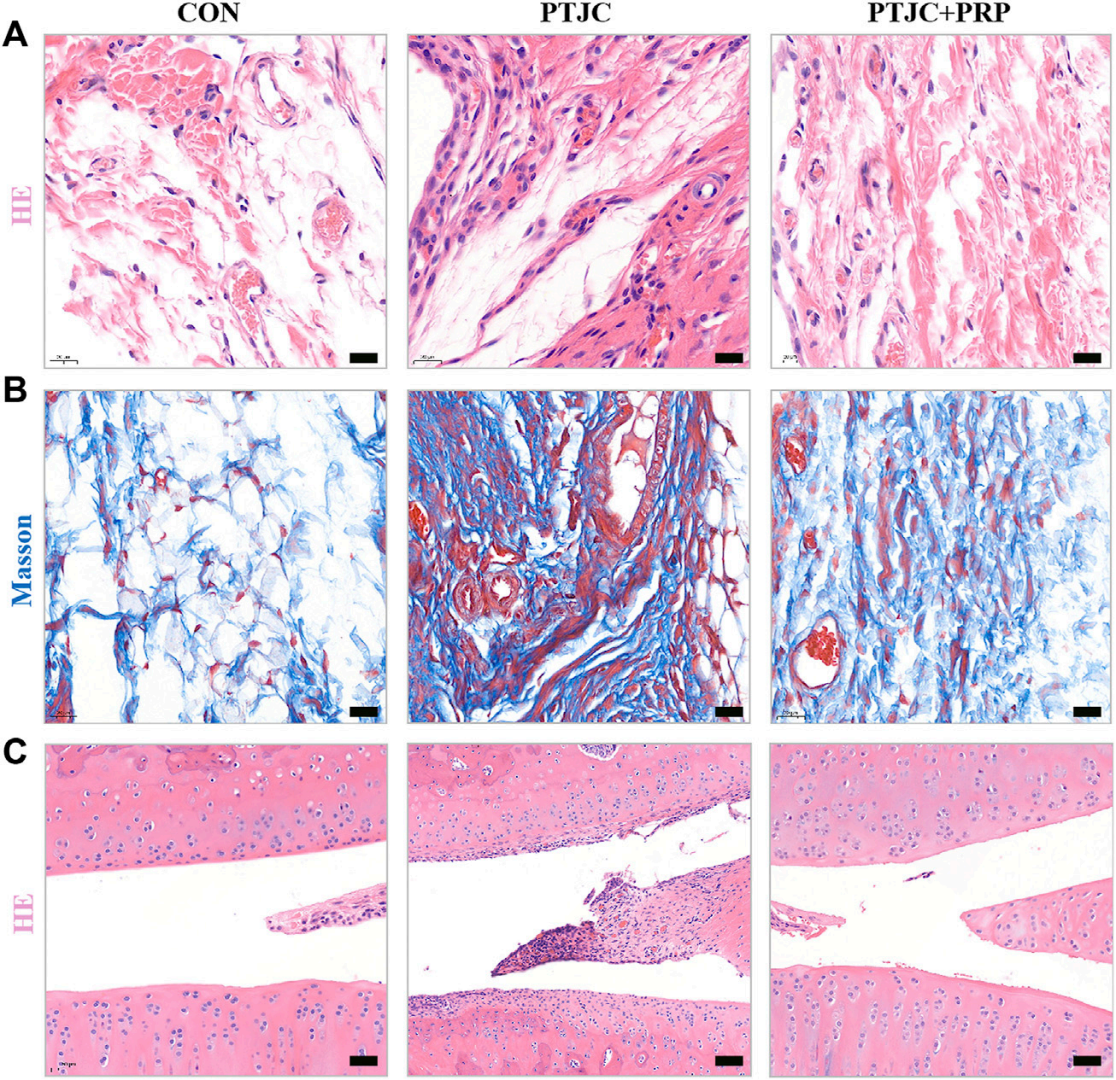
HE and Masson staining of the affected knee. **(A,B)** HE **(A)** and Masson **(B)** staining of the posterior joint capsule of the affected knee. **(C)** HE staining of articular cartilage surface. Scale bars, 50 μm.

To further evaluate the influence of PRP on PTJC, PRP/saline was injected into the lesion site. Four weeks after the injection, we performed histological analysis of the posterior joint capsule sections. The immunohistochemical staining of the experimental knees is shown in Figure 7. Histological analyses of the posterior joint capsule revealed increased numbers and density of inflammatory cells (HE) (Figure 7A), along with collagen fiber hyperplasia and disordered arrangement (Masson) (Figure 7B). These changes mirrored those found in humans and indicated that inflammation and fibrosis are closely related to PTJC. Injection of PRP at the lesion sites significantly attenuated joint capsule inflammatory cell infiltration and collagen fiber hyperplasia (Figures 7A,B). Interestingly, HE staining showed that PRP injection has the potential to delay degeneration of articular cartilage in PTJC (Figure 7C). These data indicated that PRP has anti-inflammatory, antifibrotic, and cartilage protective effects during PTJC, which may be achieved by inhibiting the production of fibrosis-related proteins and matrix degrading enzymes.

## Discussion

Our results revealed that PRP can attenuate pathological changes in joint capsule fibrosis, which may be produced by inhibiting TGF-β1/Smad2/3 signaling and downstream fibrotic marker expression in joint capsule fibroblasts during PTJC. In addition, we evaluated the effect of PRP injection on cartilage protection.

PTJC is a major musculoskeletal disease caused by trauma or surgery and is characterized by the irreversible loss of joint motion, resulting in tissue degeneration and deformity (Dunham et al., 2017). The development of PTJC is a complex process accompanied by structural changes in the joints and surrounding tissues. Among these, posterior joint capsule fibrosis is the key anatomic factor underlying PTJC (Wynn and Ramalingam, 2012; Lieberthal et al., 2015; Zhou et al., 2020). Under pathological conditions such as trauma, inflammation, collagen deposition, cytokine release, and other factors together lead to the occurrence and development of joint capsule fibrosis. Currently, there is no standard treatment for PTJC. Conservative treatment such as physical therapy and sports training, is of limited efficacy. Surgical treatment showed improved quality of life in patients with PTJC, but it is invasive, expensive, and may cause secondary damage (Dunham et al., 2017). Therefore, it is particularly important to explore more effective intervention methods for PTJC.

PRP is a plasma product extracted from whole blood and that contains platelets that release growth factors and cytokines, and has the potential to improve tissue healing and regeneration. PRP is widely used by clinicians for tissue repair and functional recovery, such as meniscal repair (Belk et al., 2020), ligament healing (Chen et al., 2018), and wound regeneration (Veronesi et al., 2018). PRP has also been shown to modulate inflammatory responses (Khatab et al., 2018). PRP can promote macrophage polarization toward the M2 phenotype (Li et al., 2016) and inhibit acute inflammation. PRP is a safe treatment with the potential to provide symptomatic benefits for hip and knee osteoarthritis (Bennell et al., 2017; Cook and Smith, 2018). Furthermore, PRP has the potential to reduce fibrosis which might be correlated with its ability to prevent myofibroblast generation (Chellini et al., 2018a). Salem et al. demonstrated that PRP could be a prospective agent for the alleviation of liver fibrosis (Salem et al., 2018). Although joint capsule fibrosis is an inflammatory and fibrosis-related disease, the effect of PRP in the treatment of joint capsule fibrosis after PTJC is unclear. Considering that animal and cell models enable research on disease pathogenesis, evaluation and treatment strategies, we developed an animal model of severe damage leading to persistent knee injury and observed ROM loss and posterior capsule fibrosis, and a series of cellular changes occurred before and after PRP intervention. This study aimed to evaluate the potential therapeutic effect of PRP in PTJC *in vitro* and *in vivo*.

TGF-β1 is considered to be predominantly involved in the process of fibrosis and plays an important role in the activation of fibroblasts, the main cellular components of the joint capsule. TGF-β1 activates fibroblasts into myofibroblasts, which express α-SMA and are considered to play an important role in the formation of contractile scars. Under physiological conditions, myofibroblasts are supposed to revert to an inactive phenotype when the temporary scar is degraded. In repeated chronic tissue damage, such as PTJC, the continuous activation of myofibroblasts promotes excessive ECM deposition, leading to the loss of ROM. TGF-β1/Smad signaling pathway plays a dominant role in the process of fibroblast differentiation. In many fibrotic diseases, TGF-β1 combines with type Ⅰ receptors, type Ⅱ receptors and relays signal transduction through phosphorylation of downstream effectors, such as Smad2/3, to promote MMP expression and collagen deposition, eventually lead to tissue fibrosis (van Caam et al., 2017; Kilari et al., 2018; Zhang et al., 2020). In our study, we showed a high phosphorylation level of Smad2/3 and up regulation of fibrosis related protein expression in the TGF-β1 group, which was significantly inhibited by the introduction of PRP *in vitro*. Similarly, PRP can also significantly inhibit the up regulation of fibrosis related proteins induced by PTJC *in vivo.* These results suggest that PRP can attenuate pathological changes of joint capsule fibrosis during PTJC, which may be implemented by inhibiting TGF-β1/Smad2/3 signaling and downstream fibrotic marker expression in joint capsule fibroblasts.

MMPs are involved in ECM remodeling (Polyakova et al., 2011). It has been reported that MMP-9 is a major regulator of the ECM in the myocardium and liver fibros and is significantly overexpressed in fibrotic myocardium of rabbits and joint capsule of frozen shoulder patients (Robert et al., 2016; Liu et al., 2017; Zhang et al., 2019b; Cho et al., 2019; Ko et al., 2019). Considering that fibrotic disease is characterized by excessive deposition of ECM and MMPs showed the ability to degrade ECM constituents, this seems to contradict the findings of our and other teams that MMP-9 expression is elevated in fibrotic diseases. Interestingly, the expression of MMP-9 seemed to dynamically change (Chen et al., 2019b; Fu et al., 2020; Li et al., 2021). In a rat model of intrauterine adhesion, Chen et al. demonstrated increased expression of MMP-9 tested in less than 2 weeks, while Li et al. showed low expression of MMP-9 after 8 weeks of modeling. Therefore, we speculated that in the early stage of fibrosis, the increased expression of MMP-9 mainly promotes the infiltration of inflammatory cells, thus promoting the process of tissue fibrosis. With the progression of fibrosis, the expression of MMP-9 gradually decreased, further intensifying the deposition of extracellular matrix.

Remarkably, we found that PRP was sufficient to inhibit TGF-β1-induced Smad2/3 phosphorylation and the expression of fibrosis markers. However, they showed no changes when cocultured with PRP alone. Similar results were seen in the research conducted by Chellini et al. (2018b). TGF-β1 is one of the most important growth factors of PRP and was also considered as one of the common regulators of the fibrotic process in many organs and tissues, which may lead to the complex effect of PRP (Giovanini et al., 2011; Moghadam et al., 2017; Pavlovic et al., 2021). The complicated effect of PRP may also be related to the effects of leucocytes in PRP. Leukocytes have the antibacterial and immunoregulatory abilities, can also stimulate the expression of cytokines, such as TNF-α and IL-1β, leading to inflammation. Devereaux et al. (2020) compared the effects between leucocyte-rich PRP (LR-PRP) and leucocyte-poor PRP (LP-PRP). Interestingly, results demonstrated that LR-PRP unexpectedly enhanced fibroblast cell proliferation and cell migration and upregulated the expression of MMPs. The exact mechanism of PRP’s complex effects still needs further exploration.

Joint-related diseases are often accompanied by cartilage damage, including joint trauma, osteoarthritis, and developmental dysplasia of the hip (Xu et al., 2020). Cartilage damage is related to the chondrocyte apoptosis, which interacts with inflammation and ECM degradation. Xue et al. (2020) demonstrated that pure-PRP was an effective non-operative treatment for inflammation and cartilage matrix loss in a rabbit model of hemorrhagic arthritis. Moussa et al. (2017) showed that PRP significantly increased proliferation and decreased apoptosis in osteoarthritic chondrocytes. Jain et al. (2019) showed that PRP could stimulate chondrocyte proliferation while downregulating the expression of COL-Ⅰ and MMP-13. Therefore, we also observed the effect of PRP on cartilage protection in the PTJC model. HE staining showed that articular cartilage was degraded after rat PTJC, and PRP injection delayed degeneration of articular cartilage. This protection effect of PRP may be achieved by inhibiting excessive inflammation, protecting chondrocyte survival and inhibiting ECM degradation.

The limitation of our study was that we did not assess the effect of PRP on the expression of inflammatory cytokines, such as TNF-α and IL-1β. Second, we did not distinguish between leucocyte-rich or leucocyte-poor PRP. Finally, we only detected the expression of fibrosis related molecules after TGF-β1 induced and PTJC models at a single point in time, but the expression of cytokines was dynamically changed. Thus, further research is needed to evaluate the antifibrotic effect of PRP in PTJC.

## Conclusion

We are the first group to reveal the antifibrotic activities of PRP at the gene and protein levels by modulating the fibrogenesis processes of joint capsule fibroblasts and a rat PTJC model. PRP markedly attenuated TGF-β1-stimulated joint capsule fibroblast activation. Crucial molecular factors, including TGF-β1, α-SMA, COL-Ⅰ, and MMP-9 that normally contribute to fibrosis were proven to be significantly reduced by the effect of PRP, which may be achieved by inhibiting the TGF-β1/Smad2/3 signaling pathway. In addition, we evaluated the effect of PRP injection on cartilage protection. Our findings provide evidence that PRP may have potential for development as an effective antifibrosis treatment strategy for PTJC in the future.

## Data Availability

The datasets used and/or analyzed during the current study are available from the corresponding author upon reasonable request.
